# Archaeol: An Indicator of Methanogenesis in Water-Saturated Soils

**DOI:** 10.1155/2012/896727

**Published:** 2012-11-22

**Authors:** Katie L. H. Lim, Richard D. Pancost, Edward R. C. Hornibrook, Peter J. Maxfield, Richard P. Evershed

**Affiliations:** ^1^Organic Geochemistry Unit, Bristol Biogeochemistry Research Centre and The Cabot Institute, School of Chemistry, University of Bristol, Cantock's Close, Bristol BS8 1TS, UK; ^2^Bristol Biogeochemistry Research Centre and The Cabot Institute, School of Earth Sciences, University of Bristol, Wills Memorial Building, Queen's Road, Bristol BS8 1RJ, UK

## Abstract

Oxic soils typically are a sink for methane due to the presence of high-affinity methanotrophic *Bacteria* capable of oxidising methane. However, soils experiencing water saturation are able to host significant methanogenic archaeal communities, potentially affecting the capacity of the soil to act as a methane sink. In order to provide insight into methanogenic populations in such soils, the distribution of archaeol in free and conjugated forms was investigated as an indicator of fossilised and living methanogenic biomass using gas chromatography-mass spectrometry with selected ion monitoring. Of three soils studied, only one organic matter-rich site contained archaeol in quantifiable amounts. Assessment of the subsurface profile revealed a dominance of archaeol bound by glycosidic headgroups over phospholipids implying derivation from fossilised biomass. Moisture content, through control of organic carbon and anoxia, seemed to govern trends in methanogen biomass. Archaeol and crenarchaeol profiles differed, implying the former was not of thaumarcheotal origin. Based on these results, we propose the use of intact archaeol as a useful biomarker for methanogen biomass in soil and to track changes in moisture status and aeration related to climate change.

## 1. Introduction 

 Methane (CH_4_) emission from soil is determined by the net balance of simultaneous *in situ *production of biogenic CH_4_ by methanogenic *Archaea* and consumption by methanotrophic *Bacteria*. In the majority of oxic soils, methanotrophy far outweighs internal CH_4_ production. The latter is assumed to be low or negligible as, although methanogens are known to survive in aerobic soils [1], they are traditionally considered strict anaerobes [2] which if present in soil are confined to anoxic microsites. Consequently, any CH_4_ produced typically is oxidised before reaching the atmosphere. Nonetheless, soils exposed to wet conditions can host substantial methanogenic communities, and in some instances act as a source of CH_4_ emissions, despite extended periods of oxygen exposure [3–6]. Consequently, we postulate that *in situ* CH_4_ production could be underestimated in such water-saturated soils, and furthermore, marginal increases in wetting caused by climate-change induced precipitation may increase the capacity of a water-saturated soil to act as a net CH_4_ source rather than a sink for atmospheric CH_4_. Thus, it is important to further understand the presence and distribution of microbial populations controlling CH_4_ production in water-saturated soil in order to assess their potential to respond to changes in soil moisture and aeration conditions, and long-term impacts of climate change.

 Membrane lipids of *Archaea* in cultures have been extensively studied and the distribution of their lipid component parts may be used for taxonomic purposes [7, 8]. Intact polar lipids (IPLs) are considered important biomarkers for living microbial biomass as polar head-groups covalently bound to the core lipid are degraded relatively quickly upon cell lysis [9]; the resultant simple lipids are therefore expected to derive primarily from fossilised material [10–12]. Phospholipids are particularly labile [9, 13], whereas glycosidic ether lipids are more resistant to degradation [14]. Archaeal IPLs differ substantially from those synthesised by bacteria, being composed of a phosphoester or glycoside polar head group ether-bound to non-polar isoprenoid glycerol diether or tetraether core lipid [15]. The core lipid archaeol is widespread in *Archaea* from a wide range of environments. Archaeal diether core lipids have been proposed as a methanogen biomarker or to estimate quantitatively methanogen biomass in natural systems. Respective studies have predominantly been restricted to highly anaerobic environments exhibiting significant CH_4_ emissions such as rice paddies [16, 17], digester sludge [18, 19], marine sediment [20], faecal material [21], permafrost [22, 23], and peatlands [24–26]. Wachinger et al. [27] observed that absolute *Archaea* cell numbers in mineral soils, calculated using the approximate ether lipid concentration 2.5 *μ*mol g^−1^ dry weight of methanogen cells [28], also reflected CH_4_ productivity. Others have instead employed the analysis of intact glycerol dialkyl glycerol tetraether (GDGT) lipid distributions as evidence of living archaeal populations in soils [29, 30].

 We postulate that archaeol present in soil predominantly originates from methanogens, and propose that archaeol abundance may serve as an indicator of methanogenic biomass. We investigate herein the concentration of archaeol in its free and conjugated forms with depth in three soils containing varying amounts of organic matter content and a high water content. Our study aims to gain new insights into methanogenic populations residing in oxic soil based upon the presence of archaeol in its free and conjugated forms, and by comparing the occurrence of archaeol to the thaumarchaeotal GDGT lipid, crenarchaeol.

## 2. Materials and Methods

**Figure 1:**
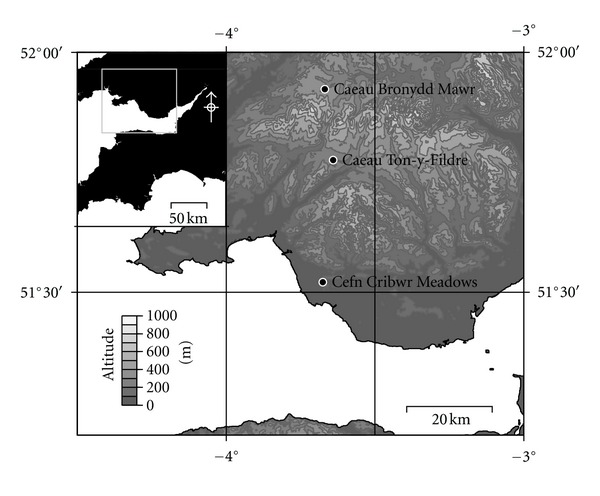
Map of Welsh sites: Caeau Ton-y-Fildre, Caeau Bronydd Mawr, and Cefn Cribwr Meadows.

 Soil cores (35 or 50 cm) were collected from three Sites of Special Scientific Interest (SSSI) managed by the Countryside Council for Wales (CCW): Caeau Ton-y-Fildre (Brecknock), Cefn Cribwr Meadows (Ogwr), and Caeau Bronydd Mawr (Brecknock) (Figure 1). Sites were selected based upon data collated in a comprehensive survey of Welsh lowland grasslands [31]. Soil properties reported by Stevens et al. [31] are provided in Table 1. Sites were selected based upon organic matter content but in general were typical of moist grassland soil in Great Britain located in areas receiving a high annual input of precipitation. Vegetation at all sites was dominated by *Molinia caerulea* (purple moor grass) with rushes, such as *Juncus acutiflorus*, also being common, and to a lesser extent sedges and smaller grasses present at Cefn Cribwr Meadows. 

 Core sections (5 cm) were frozen after collection and freeze-dried. They were then ground with pestle and mortar and 1 to 2 g was extracted using a modified Bligh Dyer solvent containing buffered water (0.05 M KH_2_PO_4_; pH 7.2) : chloroform : methanol 4 : 5 : 10 (v/v/v). An aliquot of the resulting total lipid extract (TLE) was separated into “simple lipid,” “glycolipid” and “phospholipid” fractions by silica column chromatography with chloroform : acetic acid (99 : 1, v/v), acetone and MeOH, respectively [32]. We have observed that in contrast to archaeol and acyl lipids, simple GDGTs lipids only elute in the glycolipid fraction. Phospholipid and glycolipid headgroups were cleaved by hydrolysis of ether bonds using 5% HCl in MeOH [33]. Aliquots were silylated with pyridine and *N,O*-bis(trimethylsilyl)trifluoroacetamide (BSTFA) at 70°C for 1 hr and dissolved in hexane prior to analysis by gas chromatography-mass spectrometry (GC/MS). Archaeol was quantified relative to a 1,2-di-*O*-hexadecyl-*rac*-glycerol standard. Analyses were conducted in triplicate.

 Soil pore water was analysed for acetic acid concentration by ion chromatography using a Dionex IC25 Ion Chromatograph. Acetic acid was isolated from 25 *μ*L of 0.1 *μ*m filtered pore water using two sequential IonPac HC11 analytical columns, before quantification by ion-suppressed conductivity detection. The KOH eluent flow rate was 0.2 mL min^−1^. 

 Fractions were analysed by GC/MS using a ThermoQuest Finnigan Trace GC and MS instrument equipped with a non-polar silica CP Sil5-CB column (50 m × 0.32 mm × 0.12 *μ*m) using the following temperature program: 70°C to 130°C at 20°C min^−1^, ramp to 300°C at 4°C min^−1^, and held at 300°C for 10 min. The ionisation potential was 70 eV, with the scanning range *m/z* 50–650. Selected ion monitoring (SIM) of *m/z* 130, 278, and 426 was used to enhance the sensitivity of detection of archaeol.

Semi-quantitative crenarchaeol concentrations were determined using high performance liquid chromatography/atmospheric pressure chemical ionization mass spectrometry (HPLC/APCI-MS). Analyses were performed using an Accela LC system equipped with autosampler coupled to a Thermo Scientific TSQ Quantum Access. Separation of GDGTs was achieved on an Alltech Prevail Cyano column (150 × 2.1 mm; 3 *μ*m particle size) at a flow rate of 2 mL/min, using an isocratic gradient program of hexane and hexane : isopropanol (iPA) as follows (v/v): 90 : 1 for 30 min, 87 : 73 for 5 min, 84 : 16 for 1 min, 0 : 1 for 11 min, and 9 : 1 for 13 min. APCI-MS conditions were as follows: vaporizer temperature 355°C, drying gas N_2_, and temperature 200°C, capillary temperature 280°C, corona discharge current 4 *μ*A. Crenarchaeol was detected with SIM of its protonated molecule [M+H]^+^ by mass scanning of ion *m/z* 1292 and quantified against a C_46_ GDGT standard [34]. The relative response ratio of crenarchaeol relative to the C_46_ GDGT standard is proposed to be 1 : 1, allowing semi-quantitative concentrations to be determined.

## 3. Results and Discussion 

**Figure 2:**
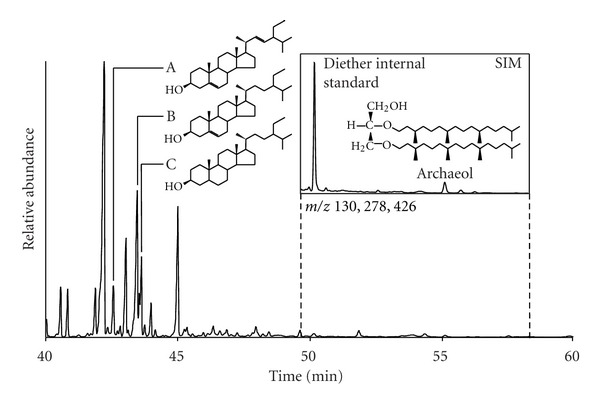
Partial total ion current (TIC) chromatogram of typical biomarker distribution at Caeau Ton-y-Fildre in simple lipid fraction. Inset: Partial chromatogram of selected ion monitoring (SIM) of *m/z* 130, 278, and 426 showing occurrence and structure of archaeol. A, 24-ethylcholesta-5,22-dien-3*β*-ol; B, 24-ethylcholest-5-en-3*β*-ol; C, 24-ethyl-5*α*-cholestan-3*β*-ol.

Biomarker distributions at all sites were consistent with previous analyses of soil. Simple lipid and hydrolysed glycolipid fractions were typically dominated by a range of higher plant-derived lipids. In addition to fatty acids, C_29_ steroids such as 24-ethylcholest-5-en-3*β*-ol, 24-ethylcholesta-5,22-dien-3*β*-ol, and 24-ethyl-5*α*-cholestan-3*β*-ol were prominent in simple lipid fractions, which also included *n*-alkanes of chain length C_25_–C_35_ with an odd-over-even predominance, and *n*-alkanols of chain length C_22_–C_34_ dominated by even-carbon numbers. The latter were also present in acid-hydrolysed glycolipid fractions, in addition to mono-, di- and *ω*-hydroxy-fatty acids. Suites of phospholipid fatty acids (PLFAs) of bacterial origin and chain length C_15_–C_24_ were observed in hydrolysed phospholipid fractions, with 16:0, 18:1*ω*7c, and 18:1*ω*9c being particularly abundant. Identification of 2,3-di-*O*-phytanyl-*sn*-glycerol (archaeol) was achieved by a combination of mass spectra in full scan mode and comparison of retention times in SIM mode with a 1,2-di-*O*-phytanyl-*sn*-glycerol standard (Figure 2). Archaeol was only detected in quantifiable amounts (detection limit 2 ng) at the Caeau Ton-y-Fildre site; Cefn Cribwr Meadows yielded trace concentrations in bound forms in the upper 15 cm whereas archaeol was not detected at Caeau Bronydd Mawr. 

**Figure 3:**
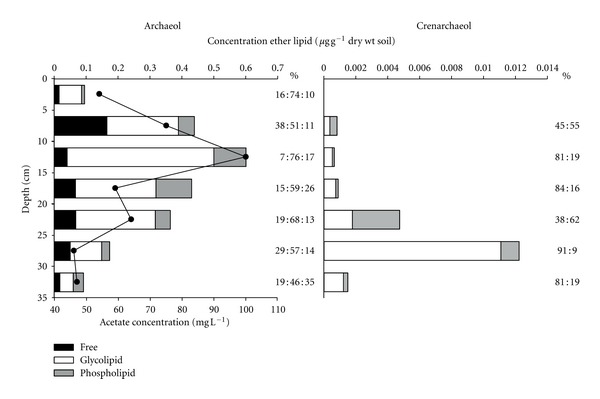
Concentrations of archaeol and crenarchaeol (*μ*g g^−1^ dry wt soil; bars; for quantification details refer to Section 2), and acetate (mg L^−1^; circles) with depth at Caeau Ton-y-Fildre. Black, white, and dark grey bars represent free lipids and ether lipids bound by glycolipid moieties and phospholipid headgroups, respectively, in the case of archaeol; for crenarchaeol, white bars include both simple lipids and glycolipids.

 Trends in free archaeol concentrations and that bound by phosphoric and glycosidic headgroups at Caeau Ton-y-Fildre are shown in Figure 3. Archaeol bound by glycolipid sugar moieties dominates, accounting for up to 76% of the total abundance (at 10–15 cm). The relatively low proportion of free archaeol suggests either: (a) prior to the polar headgroup being lost upon cell lysis, the intact archaeol is recycled into synthesis of tetraether lipids via the head-to-head condensation of phytanyl chains, consistent with biosynthetic models suggested by Nishihara et al. [35]; and/or (b) glycosidic ether lipids may form a large fraction of the fossilised pool of material due to increased resistance to degradation compared to phospholipids [14]. 

Archaeol bearing glycosidic headgroups also dominates over phosphorylated archaeol, again potentially indicating that the former derives from both living and fossil biomass. Alternatively, it could reflect preferential biosynthesis of the glycosidic form by soil-dwelling *Archaea*, as both glycolipid and phospholipid bound archaeol share the same diether precursor [36, 37]. Hence, the preference for glycolipid-bound archaeol may be due to the increased structural diversity and number of glycolipid structures synthesised by *Archaea* compared to that of phospholipid-bound core lipids [8, 36, 38]. This observation is consistent with previous studies showing that glycosidically-bound archaeal GDGTs rather than bacterial phospholipids are also the principal IPLs in sediments (e.g., [39]). 

Since glycolipid-bound archaeol may represent both fossilised and extant biomass, we suggest trends in phospholipid archaeol concentration to be representative of living methanogenic *Archaea* distributions. The Caeau Ton-y-Fildre depth profile (Figure 3) indicates an increase in total archaeol concentration from the surface to a maximum of 0.6 *μ*g g^−1^ dry wt soil at 10–15 cm, which subsequently diminishes with depth. Although this trend is mostly reflected in the profiles of the individual fractions, the phospholipid archaeol profile differs slightly, exhibiting maximum concentrations at 10–20 cm. The variance in depth profiles of phospholipid and glycosidic archaeol, particularly at 15–20 cm, suggests differing methanogen populations and/or more likely, that the distributions of living archaeal biomass differs from that of the fossilised biomass. 

Archaeol is synthesised by a wide range euryarchaeal phenotypes [40], including methanogens, extreme halophiles and thermophilic *Archaea*. The latter two are unlikely to be present in oxic soils whereas methanogens can reside in anoxic microsites [41]. Ammonia-oxidising *Thaumarchaeota*, which dominate archaeal populations in aerobic soils [29], primarily synthesise GDGTs, specifically crenarchaeol, as opposed to archaeol [42], the former being present in the majority of soils [30]. This is in accordance with the crenarchaeol depth profile differing significantly from the archaeol profile at Caeau Ton-y-Fildre (Figure 3). Concentrations of crenarchaeol increased with depth to a maximum at 25–30 cm, in contrast to archaeol which peaked at 10–15 cm, thus implying derivation from distinct archaeal populations. Although molecular approaches suggest that thaumarchaeotal depth distributions in soil differ between sites [43], similar relationships between methanogen and *Thaumarchaeota* populations have been reported in a peat [44].

The observed downcore increase of archaeol from the surface to 15 cm likely reflects the sensitivity of methanogenic *Archaea* to oxygen, although archaeol is not entirely absent in the uppermost 5 cm, potentially due to anaerobic conditions in microsites in periodically wet soil at shallow depth. Methanogens are known to survive in such conditions despite the presence of oxygen in soil pores [5, 45, 46]. In peat bogs, an abrupt increase in archaeol concentration coincides with the water table and hence inferred anaerobic conditions [24]. Although no water table is present in these soils, parallels can nonetheless be drawn, with the implication that maximum methanogen biomass will reflect the confluence of high organic substrate concentrations with the onset of sustained or extensive water saturation and development of anoxic microhabitats. Decreasing archaeol concentrations with depth likely reflect the depletion of substrates to support methanogenesis, a key factor for methanogen growth in anoxic environments [47]. Consistent with this observation, the overall trend in archaeol concentration is relatively similar to that of acetate concentrations in soil pore water (Figure 3). Although we note that acetate is not the only substrate that may support methanogenesis, it is known to dominate in the subsurface of organic-rich soils [48], and thus this agreement confirms the importance of organic substrates.

**Figure 4:**
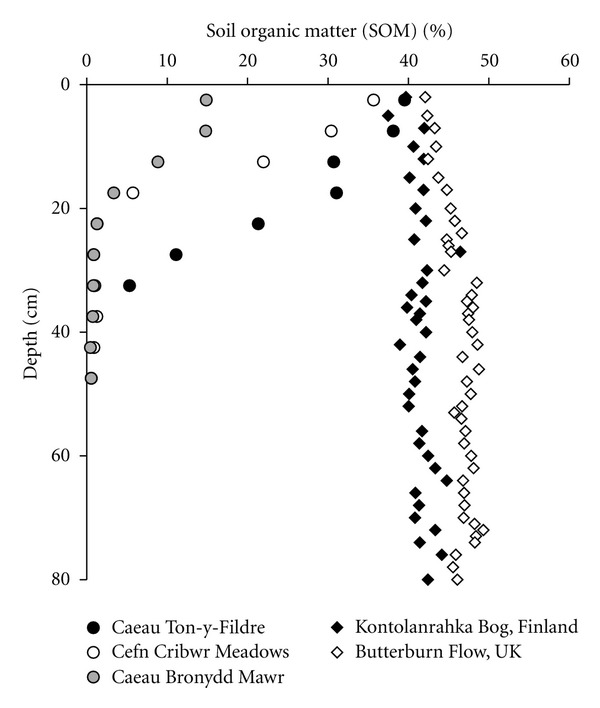
Soil organic matter (SOM) content at soil sites (dots): Caeau Ton-y-Fildre (black), Cefn Cribwr Meadows (white), Caeau Bronydd Mawr (grey); compared to peat sites [24] (diamonds): Kontolanrahka Bog, Finland (black), Butterburn Flow, UK (white).

Quantitative detection of archaeol at Caeau Ton-y-Fildre is attributed to high moisture levels and corresponding low oxygen concentrations, which will be associated with the notably greater content of soil organic matter (SOM). The abundance of SOM was highest at 39.5% at the surface of the soil, remaining high (>30%) at 20 cm depth, subsequently decreasing to 5% at 35 cm depth (Figure 4). A high moisture level exerts a primary control of methanogenic populations, and complementing this, high SOM content and heterotrophic soil respiration collectively promote the formation of anoxic microsites in soil peds, which can host anaerobic methanogens [41, 49]. Moreover, high SOM content is likely to increase substrate availability, potentially enhancing CH_4_ production [47, 50, 51]. This suggestion is supported by acetate concentrations at Caeau Ton-y-Fildre which mirror concentration trends in archaeol (Figure 3). The lower concentration of archaeol, particularly phosphorylated archaeol, at greater depths, where moisture contents remain high but acetate concentrations and SOM decrease, is evidence that both substrate availability and anoxic microsites control methanogen distribution in the soil. 

The absence or very low concentration of archaeol at the other two grassland sites is somewhat enigmatic. Cefn Cribwr Meadows exhibited SOM contents greater than 20% to 15 cm depth and then decreased markedly to 5.7%. Although SOM content was only slightly lower than at Caeau Ton-y-fildre, trace quantities of archaeol were detected in the upper part of the profile, but not below this depth. At Caeau Bronydd Mawr, where SOM content was significantly lower throughout the entire core, exhibiting a maximum of 15% carbon in the shallowest sample, archaeol was absent or below detection limit at all depths. It is possible that small differences in SOM, soil texture and consequently, water retention capacity at these two sites results in less persistent anaerobic conditions within soil peds and microsites. Thus, soil moisture content appears to manifest its impact on soil methanogen biomass in multiple, inter-related ways, via its relationship to the formation of anaerobic micro-environments, influence on organic matter preservation and possibly, substrate supply to methanogens.

**Figure 5:**
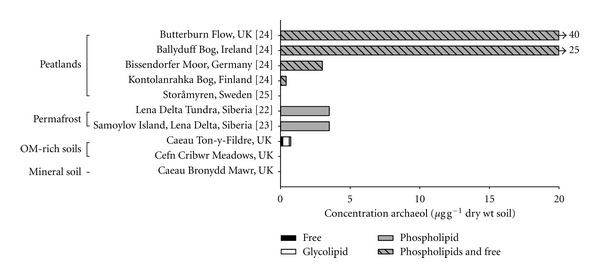
Concentration of archaeol in peat, permafrost and oxic soils (references in square brackets): free (black), glycosidic-bound (white), phospholipid-bound (grey), phospholipids and free (striped).

Figure 5 collates data from a range of sites where intact and/or free archaeol have been measured in various terrestrial environments. Data from studies reporting a combination of all archaeal diethers (e.g., hydroxyarchaeol), as opposed to solely archaeol have been excluded. Most studies of archaeol as a biomarker for methanogenic communities have predominantly focused on analyses of either free or phospholipid archaeol as tools which reflect past or modern living microbiological systems, respectively. Few studies have discussed the implications of archaeol bound by glycosidic headgroups, or all three in combination. Although glycolipids are more resistant to diagenesis than phospholipids, it is evident that a proportion of the glyco-archaeol most likely originates from living *Archaea*, since they have been observed in cultured methanogen lipid membranes. Thus, contributions from “dead” versus “living” biomass in natural systems are difficult to distinguish. 

 Regardless, our data and dataset shown in Figure 5 demonstrates that archaeol concentrations significantly increase from the SOM-lean mineral soil (Caeau Bronydd Mawr), where no archaeol is detected, to SOM-rich soils (Cefn Cribwr Meadows and Caeau Ton-y-Fildre) and permafrost, where significantly higher archaeol contents were found, and subsequently peatlands. Highest archaeol concentrations of up to 40 *μ*g g^−1^ were observed in a British ombrotrophic bog [24], despite not including glycolipids. Although variable, archaeol concentrations in peatlands (Figure 5), where high SOM is maintained throughout the profile, are generally higher than the organic-rich soils in this study, in which the SOM abundance decreases markedly below 20 cm depth (Figure 4). Concentrations observed at Caeau Ton-y-Fildre are comparable only to higher latitude peatlands, such as the Swedish and Finnish sites [24]. Moisture content, via its impact on organic matter preservation and anoxia, may exert a significant control on soil methanogen biomass and thus could influence rates of methanogenesis in various soils [52]. Corresponding trends have been observed between archaeal phospholipids and CH_4_ concentrations in permafrost [23]. It should also be noted that methanogen viability is sustained in oxic conditions, thus it cannot be excluded that biomass distributions may not reflect absolute CH_4_ production rates as oxygen suppresses methanogen capability rather than disrupting community structure [53, 54].

## 4. Conclusions

The core lipid archaeol has been investigated as a proxy for anaerobic methanogen biomass in free and conjugated forms in depth profiles of three wet oxic soils. Archaeol was detected in quantifiable concentrations at only one site, which displayed the highest soil organic matter content. Glycosidic archaeol represented a significant proportion of total archaeol, implying accumulation as a result of its recalcitrance relative to phospholipids. Alternatively, it could simply reflect that soil methanogens predominantly biosynthesise glycosidically-bound archaeol. Trends in archaeol abundance did not reflect those of crenarchaeol, representative of ammonia-oxidising *Thaumarchaeota*, confirming an origin from differing archaeal sources. High carbon contents and increased soil moisture are thought to be the interlinked factors driving observed trends in archaeol concentration, due to their association with development of anoxic microniches and substrate availability. Thus, while we acknowledge that future work should also consider tetraether archaeal intact polar lipids, we tentatively propose the use of archaeol as an indicator for methanogen biomass and consequently CH_4_ production within terrestrial soils. This provides the potential to better understand the occurrence and prevalence of methanogenesis as a result of changes in moisture, as well as their potential for CH_4_ production, and, by extension, the capacity of soils to function as a sink for atmospheric CH_4_.

## Figures and Tables

**Table 1:** Soil properties at Caeau Bronydd Mawr, Cefn Cribwr Meadows, and Caeau Ton-y-Fildre [31].

Site	Site coordinates	Texture	Dominant soil type	pH water		Exchange acidity	Cation exchange capacity	Base saturation
			(Avery 1980)	0–15 cm	15–30 cm	(meq/100 g 105° dry soil)	(meq/100 g 105° dry soil)	%
Cefn Cribwr Meadows	51 55.40′N 3 40.12′W	Loamy over clay with sandy patches	Stagnogley	5.37	5.62	1.66	9.031	81.6

Caeau Bronydd Mawr	46.57′N 3 38.46′W	Loamy over clay with sandy patches	Stagnogley	4.92	4.89	1.638	5.541	70.4

Caeau Ton-y-Fildre	51 31.28′N 3 40.5′W	Humose over sandy clay	Stagnohumic gley	5.19	5.27	1.195	8.031	85.1
